# Removal of carboxylated multi-walled carbon nanotubes (MWCNT-COOH) from the environment by *Trametes versicolor*: a simple, cost-effective, and eco-friendly method

**DOI:** 10.1038/s41598-023-43517-9

**Published:** 2023-09-26

**Authors:** Shaqayeq Asefi, Hamid Moghimi

**Affiliations:** Department of Microbial Biotechnology, School of Biology, College of Science, University of Tehran, Tehran, Iran

**Keywords:** Biotechnology, Microbiology, Environmental sciences

## Abstract

Nanotechnology has increased the release of nanoparticles into the environment, which poses a risk to human health and the ecosystem. Therefore, finding ways to eliminate these hazardous particles from the environment is crucial. This research studied the ability of *Trametes versicolor* fungi to remove carboxylated multi-walled carbon nanotubes. The study analyzed the impact of pH, MWCNT-COOH concentration, and initial fungal growth time on the removal process. The properties of the adsorbent were measured before and after the biosorption process using SEM, FTIR, and EDS techniques. The results showed that the live biomass of *T. versicolor* was more effective in removing nanoparticles than dead biomass at 30 °C and pH 7. An increase in carbon nanotube concentration from 5 to 20 mg. mL^−1^ decreased biosorption potential from 100% to 28.55 ± 1.7%. The study also found that an increase in initial fungal growth time led to higher biomass production and adsorption capacity, increasing biosorption ability for concentrations > 5mg. ml^−1^. The biosorption kinetics followed a pseudo-second-order model and corresponded most closely to the Freundlich isotherm model. The adsorption capacity of live fungal biomass to remove multi-walled carbon nanotubes was 945.17 mg. g^−1^, indicating that *T. versicolor* fungi have significant potential for removing carbon nanostructures from the environment.

## Introduction

Since the advent and progress of nanotechnology, nanoparticles with diameters of less than 100 nm have gained attention among scientists over the past few decades^1^. These nanoparticles are categorized into several groups according to their size, morphology, and chemical characteristics, i.e., carbon-based, metal, semi-conductor, ceramic, lipid-based, and polymeric nanoparticles^2^. Among all these kinds, carbon nanomaterials (CNMs) are widely used owing to their outstanding features such as high thermal conductivity, high strength, lightweight, and flexibility^2,3^.

There are several routes by which carbon nanotubes (CNTs) can spread into the environment. For example, rubbing CNT-containing tires across roads, remediation of microplastics, particularly polyethylene terephthalate (PET), and car catalytic converters can release CNTs into the soil and air. In addition, the number of CNT-containing products, such as nanocomposites, has increased over the past decades^4,5^. It is estimated that hundreds of tons of carbon nanotubes are produced worldwide each year^6^. As a result, the widespread use of these structures in nanotechnology raises concerns regarding their health and environmental impacts.

Considerable research has been conducted on carbon nanotubes, and it has been found that these recalcitrant structures exhibit toxic effects. Carbon nanotubes can induce oxidative stress by producing reactive oxygen species that can negatively affect DNA, damage cell membranes and proteins^7^, and induce the transcription of genes related to cell apoptosis^8,9^. Furthermore, they pose risks to human health, organisms, and microorganisms in an ecosystem. CNMs are hazardous to human health, and toxicological studies have suggested they pose similar risks to asbestos^10^. Because of their small size and low weight, these structures can enter the lungs through the air and induce inflammatory responses, eventually leading to lung cancer^11,12^. In addition, they are cytotoxic to human skin cells and can cause skin cancer in the long term^9^. The release of wastewater containing CNTs has the potential to harm aquatic life. Since they are insoluble in water, they tend to accumulate in sediment, which can be toxic to benthic animals in rivers. Some researchers have studied the toxicity of CNTs to aquatic organisms and found that an increase in SWCNTs concentration can have a negative impact on the development of *Artemia salina* in seawater, leading to larval mortality^13^. Studies on the effect of SWCNTs on zebrafish have also shown that CNTs can stimulate the production of neurotransmitters that affects the physiology and behavior of organism^14^. Furthermore, studies have indicated that CNTs can pose a risk to soil organisms such as plants. For instance, MWCNTs have been found to increase the generation of reactive oxygen species in spinach, resulting in growth reduction, necrotic lesions of leaf cells/tissues and changes in root and leaf morphology^15^. In addition, CNMs are toxic to microorganisms. They can reduce or hinder their growth or negatively affect their diversity in an ecosystem. Therefore, based on these negative effects of carbon nanostructures on humans and the environment, it is necessary to find a practical solution for removing these structures from wastewater before entering the environment^12,16–18^.

Generally, several physicochemical (membrane processes, electrocoagulation, ionic exchange, coagulation-flocculation, precipitation, etc.) and biological methods (biodegradation and biosorption) are suggested to remove pollutants in wastewater treatment^19^. Biological treatments have gained huge attention among all these treatment methods due to their sustainability and low-cost^20^. Removal methods for eliminating CNTs from entering the environment are categorized into non-biological degradation (using UV, Fenton, H_2_O_2_) and biological degradation (using myeloperoxidase, manganese peroxidase, lignin peroxidase, and lactoperoxidase enzymes)^21–24^. However, these methods have some drawbacks, including low efficiency, inability to complete the degradation of these structures, and longtime degradation process. Therefore, this investigation investigated the applicability of biosorption methods as an alternative to other conventional processes.

The biosorption mechanism involves interactions between biosorbents and pollutants based on physical, chemical, and metabolic processes^25^. Biosorption mechanisms vary depending on the cell metabolism and the location where pollutants are removed from the environment. Biosorption based on cell metabolism can be divided into metabolism-dependent and non-metabolism-dependent mechanisms. Whereas biosorption based on the location of removed particles is categorized into extracellular accumulation/precipitation, cell surface sorption/precipitation, and intracellular accumulation. However, it should be noted that these processes are not mutually exclusive and can occur simultaneously^26^. Bacteria, yeasts, algae, and fungi are among the common biosorbents^27^. However, the distinct properties of fungi, such as high biomass production and presence of glucan, chitin, and mannoproteins in their cell wall as well as availability and chemical stability, have positioned them as one of the most commonly used biosorbents for wastewater treatment^28,29^. Furthermore, the presence of amino, carboxyl, thiol, and phosphate groups in their cell walls enable them to attach to pollutants and eliminate them from the contaminated environment^30^. Both live and dead fungal biomasses can be utilized for the biosorption process. The biosorption mechanisms employed by dead biomass are typically classified as passive or non-metabolic processes. Conversely, live biomass plays an integral role in biosorption through metabolic-dependent processes that operate with passive mechanisms^31^. So far, many studies have been done to evaluate live/ dead fungal biomass such as *Funalia trogii*^32^, *Aspergillus niger*^33^, *Aspergillus foetidus*^34^, *Phanerochaete chrysosporium*^32^, and *Trichoderma harianum* for removing various pollutants such as heavy metals and dyes.

In this study, for the first time, we investigated the biosorption ability of carboxylated multi-walled carbon nanotubes using *T. versicolor*. In this context, we aimed to evaluate the potential of live and dead fungal biomass to remove MWCNT-COOHs from the solution. The effects of MWCNT-COOH concentration, initial pH, and initial fungal growth time on the biosorption potential were also determined. Finally, FTIR, EDS, and SEM analyses were used to examine the biosorbent morphology, biosorption of MWCNT-COOHs, and determination of the functional groups. These findings add to our understanding of fungal cell abilities as biosorbents to remove pollutants from the environment and introduce an environmentally friendly method for removing nanostructures.

## Materials and methods

### Biosorbent preparation

*Trametes versicolor* (fungal strain) was supplied by the Environmental Biotechnology Laboratory of the University of Tehran. According to articles on biosorption by fungal strains, different culture media were used to investigate the biosorption process of different fungal species. Therefore, three different media were tested to find a suitable culture medium where *T. versicolor* could grow and produce high amounts of biomass. In this case, *T.versicolor* was cultivated on PDB (Potato infusion 4g.L^−1^, -Dextrose 20 g.L^−1^), Sucrose-Yeast Extract (Sucrose 30 g.L^−1^, Yeast extract 10 g.L^−1^), and Sucrose-Yeast Extract-Salt (Sucrose 100 g.L^−1^, KH_2_PO_4_ 0.5 g.L^−1^, KCl 0.025 g.L^−1^, NaNO_3_ 1.5 g.L^−1^ MgSO_4_.7H_2_O 0.025 g.L^−1^, Yeast extract 1.6 g.L^−1^). Circular plugs of *T. versicolor* with a diameter of 1 cm were cut from the PDA and inoculated into the culture medium. The fungal biomass was harvested after 7 days of incubation at 30 °C and 160 rpm on a thermostat shaker, washed, and treated with distilled water. The fungal biomass was dried at 60 °C for 24 h, and the amount of biomass was weighed and analyzed.

### Biosorption experiment

The biosorption studies were performed using batch techniques. The adsorption process on the fungal biomass was analyzed by measuring the initial weight of MWCNT-COOH [MWCNTs functionalized with COOH (> 95%, OD: 20–30 nm) with elemental contents of Ni (0.93%), Cl (0.45%), C (98.39%), and Fe (0.23%) provided from US Research Nanomaterials] and comparing it with the residuals remaining after biosorption. Circular fungal plugs with a diameter of 1 cm were inoculated into 100 mL Erlenmeyer flasks with 20 mL culture media containing 5 mg. mL^−1^ MWCNT-COOH concentration. Adsorption was carried out at 30 °C, and the batch media were maintained in a thermostatic shaker with a continuous agitation speed of 160 rpm for 7 days. After the cell growth and biosorption processes, fungal pellets were collected using tweezers, and other contents of the flasks were centrifuged at 5000 rpm for 15 min. The supernatant was removed, and the remaining MWCNT-COOH was dried at 60 °C for 24 h. Finally, the number of carbon nanotubes remaining in the flasks was deducted from the initial amount to obtain the nanocarbons adsorbed by the fungal pellets. % Biosorption and Q were calculated with Eqs. (1) and (2).

1 $$ {\text{Biosorption}}\,\% \, = \,\left[ {\left( {{\text{C}}_{{\text{i}}} - {\text{C}}_{{\text{f}}} } \right)/{\text{C}}_{{\text{i}}} } \right]\, \times \,{1}00, $$

2 $$ {\text{Q}}\, = \,\left( {{\text{C}}_{{\text{i}}} - {\text{C}}_{{\text{f}}} } \right)\, \times \,{\text{V}}/{\text{m}}{.} $$

In this equation, C_i_ is the amount of initial nanocarbon (mg. mL^−1^); C_f_ is the amount of remaining nanocarbon after the biosorption process (mg. mL^−1^); V is solution volume (mL); m is biosorbent mass (g); and Q is adsorption capacity (mg. g^−1^).

### Optimization of biosorption experiments

#### Biosorption by live and dead fungal biomass

The biosorption experiment was performed under batch conditions with both live and dead fungal biomass to determine whether the biosorption process is a metabolic-dependent or metabolic-independent mechanism. Two methods of analysis were used to prepare the dead biomass. The first method involves inoculating fungal pellets into an Erlenmeyer flask containing PDB culture media and allowing them to grow for 17 days. The fungal pellets were extracted, washed twice with distilled water, and dried for 24 h at 60 °C. For the second method, the fungal biomass was extracted after growing for approximately 17 days and autoclaved for 20 min at 121.5 °C. Subsequently, the dead fungal biomass was incubated in sterile distilled water containing 5 mg. mL^−1^ MWCNT-COOH. Finally, the amount of carbon nanotube biosorption was analyzed.

### Effect of pH on Biosorption

The pH of the solution was determined to be an effective parameter in carbon nanotube adsorption. To optimize the conditions, acidic and basic pH values ranging from 3 to 11 were investigated. Experiments on removing carbon nanotubes were conducted under the same conditions described above.

### Effect of initial carbon nanotube concentration on biosorption

To examine the impact of the initial concentration of MWCNT-COOH on the adsorption mechanism, the fungal biomass was placed in four flasks containing 5, 10, 15, and 20 mg. mL^−1^ carbon nanotubes. Circular plugs of fungal mycelia with a diameter of 1 cm were inoculated into the assay medium, including the required quantity of carbon nanotubes. The biosorption ratio was determined by measuring the reduced carbon nanotube content after 7 days of incubation.

### Effect of initial fungi growth time on biosorption

To determine the suitable time for inoculation of carbon nanotubes into the culture media and the effect of biomass concentration on biosorption, the addition of carbon nanotubes to the culture media was analyzed at different time intervals, including the same time of fungal inoculation, 2 days after fungal growth, 4 days after fungal growth, and 6 days after fungal growth. Subsequently, the biosorption rate and fungal biomass were determined.

### Biosorption isotherms and kinetics calculation

The optimal interaction period between MWCNTs and biosorbents was determined using kinetic analysis. Kinetic studies were performed by growing fungal pellets for 4 days and adding 10 mg. mL^−1^ carbon nanotubes to the culture medium. The flasks were placed in a shaking incubator at 160 rpm and 30 °C. Samples were collected from the flasks every 12 h at periodic intervals. Finally, nanocarbon removal was estimated by measuring biosorption in the medium. The kinetics of adsorption for the removal of MWCNT-COOHs from the medium was measured using the pseudo-first-order (PFO) and pseudo-second-order (PSO) model equations given in Eqs. (3) and (4), respectively^35,36^.

3 $$ {\text{ln}}\left( {{\text{q}}_{{\text{e}}} - {\text{q}}_{{\text{t}}} } \right)\, = \,{\text{lnq}}_{{\text{e}}} \, - \,{\text{k}}_{{1}} .{\text{t,}} $$

4 $$ {\text{t}}/{\text{q}}_{{\text{t}}} \, = \,{1}/\left( {{\text{k}}_{{2}} {\text{q}}_{{\text{e}}}^{{2}} } \right)\, + \,{\text{t}}/{\text{q}}_{{\text{e}}} , $$

where k_1_ is the adsorption rate constant (h^−1^) for PFO, k_2_ is the rate constant (mg. g^−1^.h^−1^) for PSO, q_e_ is the adsorption capacity (mg. g^−1^), and t is time (h).

Different concentrations of MWCNT-COOH, including 20 mg. mL^−1^, 15 mg. mL^−1^, and 10 mg. mL^−1^, were studied to determine the adsorption isotherm model for MWCNT-COOH. The adsorption of MWCNT-COOH onto the fungal biosorbent was simulated using Langmuir and Freundlich isotherm models given in Eqs. (5) and (6), respectively^36,37^.

5 $$ {1}/{\text{q}}_{{\text{e}}} \, = \,{1}/\left( {{\text{k}}_{{{\text{l}}.}} {\text{q}}_{{{\text{m}}.}} {\text{C}}_{{\text{e}}} } \right)\, + \,{1}/{\text{q}}_{{\text{m}}} , $$

6 $$ {\text{ln q}}_{{\text{e}}} = {\text{ ln k}}_{{\text{f}}} + { 1}/{\text{n ln C}}_{{\text{e}}} , $$

where k_f_ is the constant of the Freundlich isotherm and 1/n is parameter relates to the biosorption intensity, k_l_ is the constant of the Langmuir isotherm (mL.mg^−1^), q_m_ is the maximum amount of adsorbed nanocarbon (mg. g^−1^), and n is the adsorption constant.

### Characterization techniques

#### SEM–EDS analysis

Scanning Electron Microscopy (SEM) in conjunction with Energy Dispersive X-Ray Analysis (EDS) at 25.0 kV and × 1000 and × 5000 magnification (Zeiss, Sigma 300) was used to characterize the fungal biosorbent before and after the biosorption processes. The surfaces of the unloaded and nanocarbon-loaded fungal biosorbents were examined using this technique. The dried fungal biomass before and after treatment with 5 mg. mL^−1^ nanocarbon at an optimum pH of 7.0 and 30 °C was fully prepared for SEM–EDS studies.

#### Infrared spectral analysis

Using an FTIR spectrometer, the spectra of unloaded and nanocarbon-loaded fungal biomass were collected. The powdered and dried samples were analyzed in the 4000–400 cm^−1^ spectral interval at 4 cm^−1^ resolution.

### Statistical analysis

The experimental data were statistically analyzed using SPSS v26. Each experiment was performed with three replicates. The Shapiro–Wilk test examined the normal distribution of the collected findings and revealed that all data were normally distributed. Error bars represent the standard deviation in the figures. Finally, ANOVA was followed by a post hoc Tukey’s test to assess the difference between means. Statistical significance was set at p < 0.05.

## Results and discussion

### Comparing fungal growth in different culture media

Biomass is the key element for the biosorption process^31^. The fungal growth potentials of the three culture media were investigated to determine a suitable culture medium for biomass production. Among the culture media analyzed in this experiment, Potato-Dextrose (2.25 ± 0.52 g. L^−1^) medium produced the most biomass, whereas the Sucrose–Yeast Extract (1.51 ± 0.36 g. L^−1^), and Sucrose-Yeast Extract-Salt (0.46 ± 0.07 g. L^−1^) produced the least biomass; therefore, Potato-Dextrose medium was selected as the suitable culture medium for biosorption analysis.

### Comparing dead and live biomass for carbon nanotubes removal

The fungal biomass was examined under both situations to compare dead and live biomass. According to the results, live biomass was chosen for further testing on MWCNT-COOH removal because it showed 100% carbon nanotube removal at 30 °C and pH 7 after 7 days of incubation with 5 mg. ml^−1^ carbon nanotube concentration. Therefore, it can be concluded that removing carbon nanotubes from the environment by *T. versicolor* is a metabolic-dependent process. This means that only live cells can facilitate this type of biosorption. It is commonly linked to a microorganism’s active defense system, which responds to toxic pollutants. However, this process is not instantaneous as it involves the microorganism’s reaction time^26^. Utilizing living microorganisms for pollutant removal is advantageous because it avoids the challenges of biomass cultivation, harvesting, drying, processing, and storing before use^38^; therefore, they are more economical than dead biomass. There have also been reports on removing environmental pollutants using live biomass. For example, the living biomass of *Pichia pastoris* was used as a suitable procedure to remove AG1 and RR11 from wastewater. This living biomass showed a 55.35% removal capacity at 30 °C and pH 2^39^. A similar study on biosorption using live biomass was performed by Cheng et al*.,* in which the biosorption of Cd^2+^ by live biomass of *Chlorella vulgaris* algae showed a 95.2% of removal ability^40^. The potential of live and dead *T. versicolor* fungi was analyzed to remove synthetic dyes, such as Congo Red and Astrazon Black, from the environment. According to these reports, *T. versicolor* live fungi biomass can remove 80–100% of dyes from the environment, while this removal potential decreased to 50–70% in dried and dead biomass^41,42^ The potential of *T. versicolor* to remove different pollutants from the environment is summarized in Table 1.

**Table 1:** Comparing studies that have utilized *T. versicolor* cells as biosorbent.

Biosorbate	Experimental condition	Concentration	Q_m_ (mg. g^−1^)	Biosorption (%)	Reference
Astrazon black	Temperature 30 °C	100 mg. L^−1^	N/A	20–90	^41^
Cd (II)	Temperature 20 °C; pH 6	30–700 mg. L^−1^	124	68–84	^43^
Congo red	Temperature 30 °C pH 7	10–50 mg. L^−1^	38.91	N/A	^42^
Congo red	Temperature 27 °C	10–50 mg. L^−1^	52.91	87.9–100	^44^
Bisphenol-A	Temperature 25 °C	0.05 mg. L^−1^	N/A	76	^45^
MWCNTs-COOH	Temperature 30 °C	5–20 mg. mL^−1^	1000	28.55–100	In this study

### Effect of pH on fungal growth and biosorption

Changes in pH can affect the interaction between the adsorbate and adsorbent. Factors such as the activity of functional groups in biomass, the chemistry of adsorbate in the solutions, and the net charge on the surface of sorbent cells are all affected by pH levels^46^. At higher pH values, the surface of fungal biosorbents becomes negatively charged due to deprotonation. In contrast, in lower pHs, positive charges are widespread on the surface of the biomass as a result of porotonation^42,47^. The effect of this factor on the evolution of biosorption by *T. versicolor* was analyzed in the pH range of 3–11. Our results demonstrated that an increase in pH levels from acidic to neutral pH (7) will enhance biosorption.

**Figure 1:**
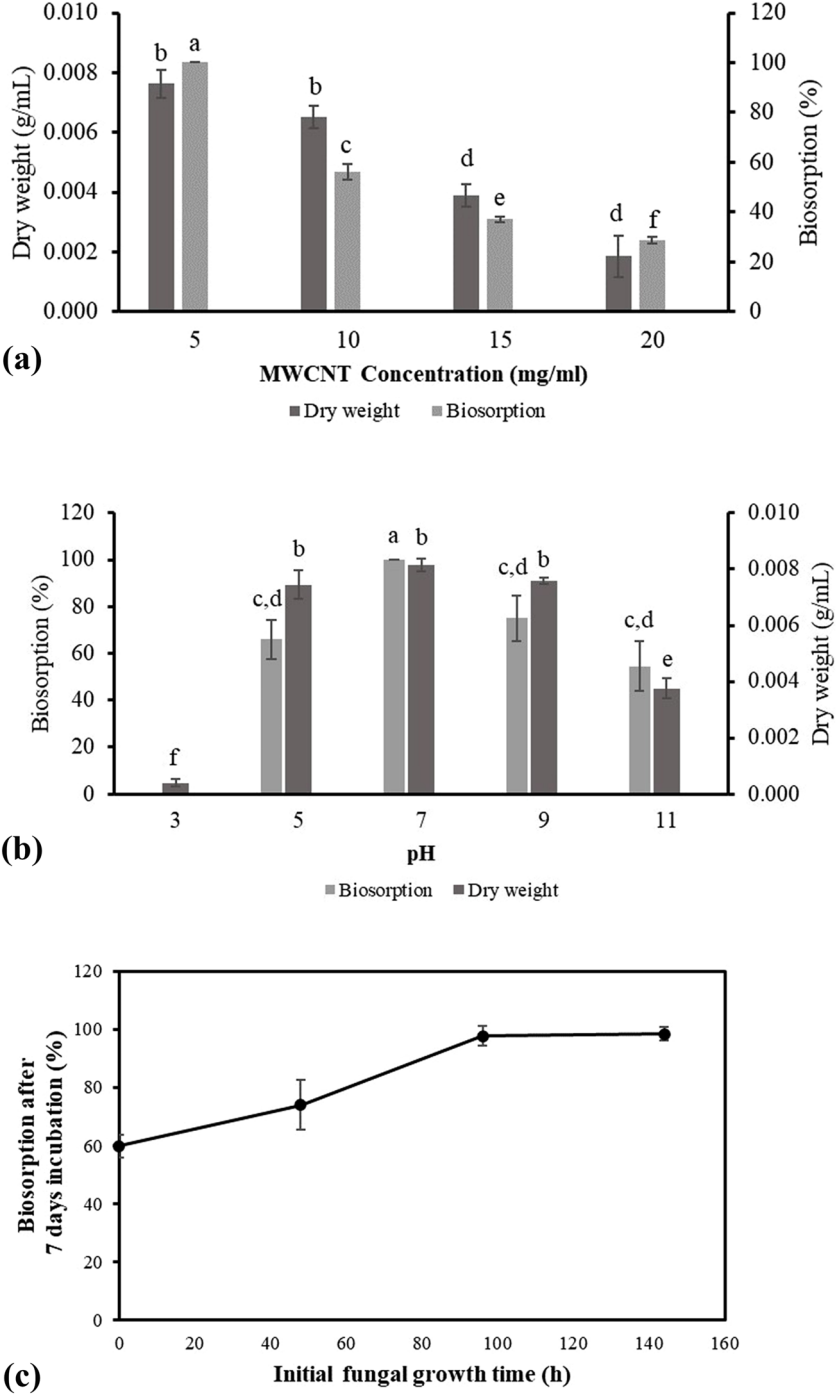
Effect of different experimental condition on biosorption of MWCNT-COOH. (**a**) Effect of pH on fungi growth and biosorption; (**b**) Effect of MWCNT-COOH concentration on fungi growth and biosorption; (**c**) Effect of initial fungal growth time on biosorption. Experiments were performed with three replicates. Each value shown as mean ± S.D. Different letters indicate significant differences (p < 0.05).

Meanwhile, with increasing pH of the batch solution toward alkalic conditions, the biosorption will be decreased. In addition, MWCNT-COOHs have a negative charge in the pH range of 2–10^48^, which suggests that biosorption by *T. versicolor* should be increased with a reduction in pH value. However, the result of this study indicated that this fungus eliminates MWCNT-COOHs in high levels at pH 7. This highlights that MWCNT-COOHs removal by *T. versicolor* fungi is not generally influenced by the net charge of the adsorbent surface; in contrast, it is directly related to the fungal growth ability. As depicted in Fig. 1a, the fungi can produce high biomass levels at pH 7, consequently increasing adsorption capacity and percentage.

Moreover, the biosorption of carbon nanotubes at pH 5 and 9 is greater than the biosorption rate at highly acidic and alkaline pH values such as pH 3 and 11. Furthermore, a comparison of the growth rate and biomass production at these pH values showed that the fungi had the best growth at pH values of 5 and 9 compared to pH values of 3 and 11, which is a good explanation for high biosorption rate at these pH values. In conclusion, due to the fact that biosorption process in this study relies on metabolism, the pH level has a significant impact on the growth of fungi. When the pH is favorable, fungi can grow and produce a large amount of biomass, resulting in high potential for adsorption. A similar pattern of results was obtained in a study on removal of Direct Blue 1 and Direct Red 128 by *T. versicolor*. The native biomass of this fungi showed a significant adsorption capacity at neutral pH, while at high acidic and alkalic condition the adsorption capacity was reduced, significantly^49^. Recently, similar conclusion was reached in a study on biosorption of copper to *Pinus radiata* sawdust, in which pH = 7 was selected as the optimum pH with 93.4% adsorption efficiency^50^.

### Effect of initial carbon nanotube concentration on fungal growth and biosorption

As discussed previously, nanomaterials are toxic to microorganisms and can hinder or reduce their growth rates. In addition, they can affect the enzyme and metabolite production by microorganisms. An increase in CNT concentration directly affected biomass production and the biosorption ability of fungi. The maximum and minimum carbon nanotube removal was observed at 5 mg. ml^−1^ and 20 mg. ml^−1^, with 100% and 28.55 ± 1.7% removal performance, respectively. The results depicted in Fig. 1b indicated that as carbon nanotube concentration increases, the ability of fungi to remove MWCNT-COOH decreases according to the effect of carbon nanotubes on fungal growth, consequently reducing the removal ability. This concept is because the high concentrations of CNTs are most likely inhibiting the growth of fungi by preventing the production of new hyphae and/or lysis of cells, causing a reduction in the contents of protein, lipid, and polysaccharides^51^.

Interestingly, according to the results of this study, it can be inferred that carbon nanotube concentrations of up to 5 mg. mL^−1^ do not affect fungal growth and adsorption potential. In contrast, high concentrations of carbon nanotubes have toxic effects on fungal growth, hindering microbial growth and reducing the removal potential of fungal biomass. This result is consistent with other reports on the effects of nanoparticles on microorganisms. An instance of this is the investigations conducted on *Phanerochaete chrysosporium*. This white-rot fungus demonstrated that an increase in GO concentration leads to a decrease in fungal growth rate. Moreover, it was observed that MWCNT-COOH did not have any inhibitory effect on fungal growth when present in concentrations ranging from 0–1 mg.ml^−1^^52,53^. The inhibitory effects of nanoparticles on fungi are shown in Table 2.

**Table 2:** Comparison of the inhibitory effects of nanoparticles on fungi.

Nanoparticle	Concentration	Fungi	Inhibitory concentration	References
Quantum dot	0–8 mg. L^−1^	*T. versicolor*	4–8 mg. L^−1^	^51^
SDS/DDAB	0–6.8 g. L^−1^	*T. versicolor*	3.2–6.8 g. L^−1^	^51^
Graphene	0–4 mg. mL^−1^	*P. chrysosporium*	4 mg. mL^−1^	^52^
MWCNT-COOH	0–1 mg. mL^−1^	*P. chrysosporium*	No inhibitory effect	^53^
MWCNT-COOH	5–20 mg. mL^−1^	*T. versicolor*	> 5mg.mL^−1^	In this study

### Effect of initial fungal growth time on biosorption

The effectiveness of carbon nanotubes in fungal biomass biosorption was previously found to be low at high concentrations. This study aimed to investigate the impact of fungal growth time on carbon nanotubes' biosorption performance. Figure 1c displays that growing fungal biomass in a solution without carbon nanotubes for 4 and 6 days and then introducing a high concentration of carbon nanotubes to the previously grown culture media would increase the removal potential at higher concentrations. For instance, the fungal biomass’s potential to remove 10 mg.ml^-1^ of carbon nanotubes from the environment was 56.1 ± 4.03%. However, this amount increased to 100% in fungal biomasses grown for 4 and 6 days. Thus, it can be inferred that increased biomass will enhance the adsorption capacity and fungi's ability to remove carbon nanotubes at higher concentrations. In another perspective, it can be inferred that an increase in initial fungal growth time enhances initial biomass dosage in the experimental batch condition, increasing the surface area of sorbent and the availability of more binding sites^54^. A similar study on the biosorption of Cu^2+^ by *T. versicolor* fungi revealed a similar conclusion that an increase in biomass dosage would significantly improve biosorption from approximately 20–80%^55^.

### Isotherm and kinetic study

**Figure 2:**
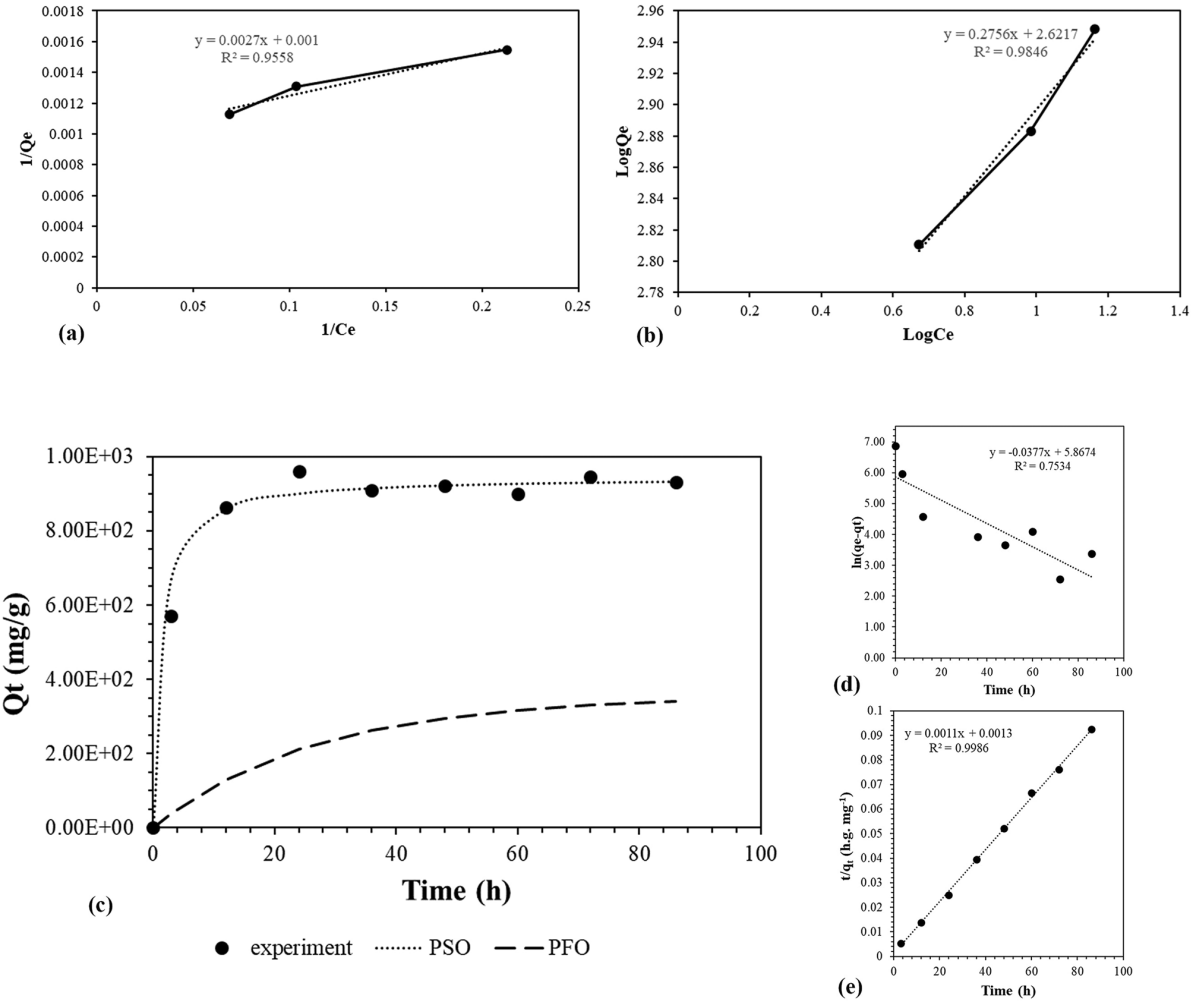
Langmuir (**a**) and Fruendlich (**b**) isotherm models; Non-linear kinetic model (**c**); Pseudo-first-order (**d**); and Pseudo-second-order models (**e**) of MWCNT-COOH biosorptuin by *Trametes versicolor* fungi. Experiments were performed with three replicates.

The adsorption isotherm demonstrates the relationship between the adsorbate and adsorbent^56^. This work investigated the interaction of carbon nanotube adsorbate and *T. versicolor* adsorbent using the Langmuir and Freundlich isotherms. The Langmuir model postulates that adsorption is monolayer and occurs at specified homogeneous sites. In contrast, the Freundlich isotherm accounts for the exponential spread of binding sites with a multilayer pattern around the adsorbent^56^. Figure 2a and b depict the Langmuir and Freundlich isotherm diagrams, respectively. The Langmuir parameters can be determined by plotting 1/q_e_ against 1/Ce, while the values of Freundlich isotherm parameters were obtained by plotting logq_e_ against logCe^57^. The isotherm analysis results showed that the biosorption process best fits the Freundlich isotherm model, with a correlation coefficient 0.98 and k_f_ = 13.7588, indicating that MWCNT-COOH adsorption on fungal surfaces is heterogeneous and multilayer. In the Freundlich isotherm, 1/n is the adsorption intensity, and n > 1 indicates an efficient interaction between the adsorbate and the adsorbent; accordingly, n = 3.63 in this report illustrates an efficient interaction between MWCNT-COOH and the surface of the fungi. The maximum capacity of the biosorbent was 1000 mg. g^−1^. Interestingly, the same results were obtained in a study on biosorption of SiO_2_-FITC using heterotrophic wastewater biomass in which the data indicated that the mechanism of biosorption in this system was also well-fitted with Freundlich isotherm model with a regression coefficients of 0.979^58^.

Time is another important factor in biosorption. The kinetic process has been determined by the adsorbate transfer to the adsorbent surface and the formation of a surface film, diffusion into particles, and diffusion into the surface pores^56^. Pseudo-second-order (PSO) and Pseudo-first-order (PFO) kinetic models are often employed. Figures 2c displays the non-linear model, and Fig. 2d and e depict the linearized model of the pseudo-first- and pseudo-second-order models of kinetics. The correlation coefficient was used to measure the fitness of biosorption for the kinetic models and determine the appropriate model. Based on the correlation coefficient value, it was hypothesized that the adsorption procedure adhered to the pseudo-second-order model with R^2^ = 0.99, q_e_ = 945.176 mg.g^−1^, and k = 0.00087 g. mg^−1^ h^−1^.

In a similar study, the removal of dyes with live biomass of *Rigidoporus vinctus* showed that the kinetic removal of Congo Red and Methylene Blue dyes by this white-rot-fungus also followed a pseudo-second-order model with a qe = 14.9 and 16.6 mg. g^−1^, and k = 0.001 and 0.0006 g/mg/h, respectively^57^. The parameters derived from the isotherm and kinetic models are summarized in Table 3.

**Table 3:** The parameters for isotherm and kinetic models.

Isotherm model							
Langmuir				Fruendlich			
k_l_	q_m_	R^2^		k_f_	1/n	R^2^	
0.36411	1000	0.95		13.759	0.2756	0.98	

Kinetic model							
Pseudo-first-order				Pseudo-second-order			
Equation	q_e_	R^2^	k_1_	Equation	q_e_	R^2^	K_2_
y = − 0.0377x + 5.8674	353.32	0.75	− 0.0377	y = 0.0011x + 0.0013	945.17	0.99	0.00087

*k_l_ (mL.mg^−1^), q_m_ (mg.g^−1^), q_e_ (mg.g^−1^), k_1_ (h^−1^), k_2_ (g.mg^−1^.h^−1^).

### Biosorbent characteristic

#### FTIR analysis

A systematic investigation was conducted to elucidate the adsorption mechanism. The different infrared bands the adsorbed carbon nanotubes produced were analyzed by capturing the FTIR spectra in the 400 to 4000 cm^−1^ range.

FTIR peak shift of more than 4 cm^−1^ is considered significant^59^. Changes in FTIR picks are categorized into (1) removal of peaks related to C-N and C = O functional groups, (2) changing the intensity of peaks associated with N–H and O–H functional groups, and (3) creation of new peaks associated with C–C, C-H, and C–Cl. According to the FTIR results, considerable changes in the intensity of peaks associated with N–H of primary amines and O–H functional groups from 1625.67 cm^−1^ and 3419.34 cm^−1^ shifts to 1619.96 cm^−1^ and 3425.60 cm^−1^, respectively, indicates the significant contribution of hydroxyl and amine groups in the biosorption of MWCNT-COOH to fungal mycelium pellets. Hydroxyl and amine groups are the most important functional groups in polysaccharides and protein structures in the fungal cell wall^60^. The change in the peaks obtained from these two groups indicates the interaction and bonding of MWCNT-COOH with these two functional groups. In addition, the formation of new peaks related to the C–H, C–C, and C–Cl groups that exist in the structure of multi-walled carbon nanotubes in the FTIR analysis of fungi and nanocarbon confirmed the interaction of these structures with the fungal cell wall.

**Figure 3:**
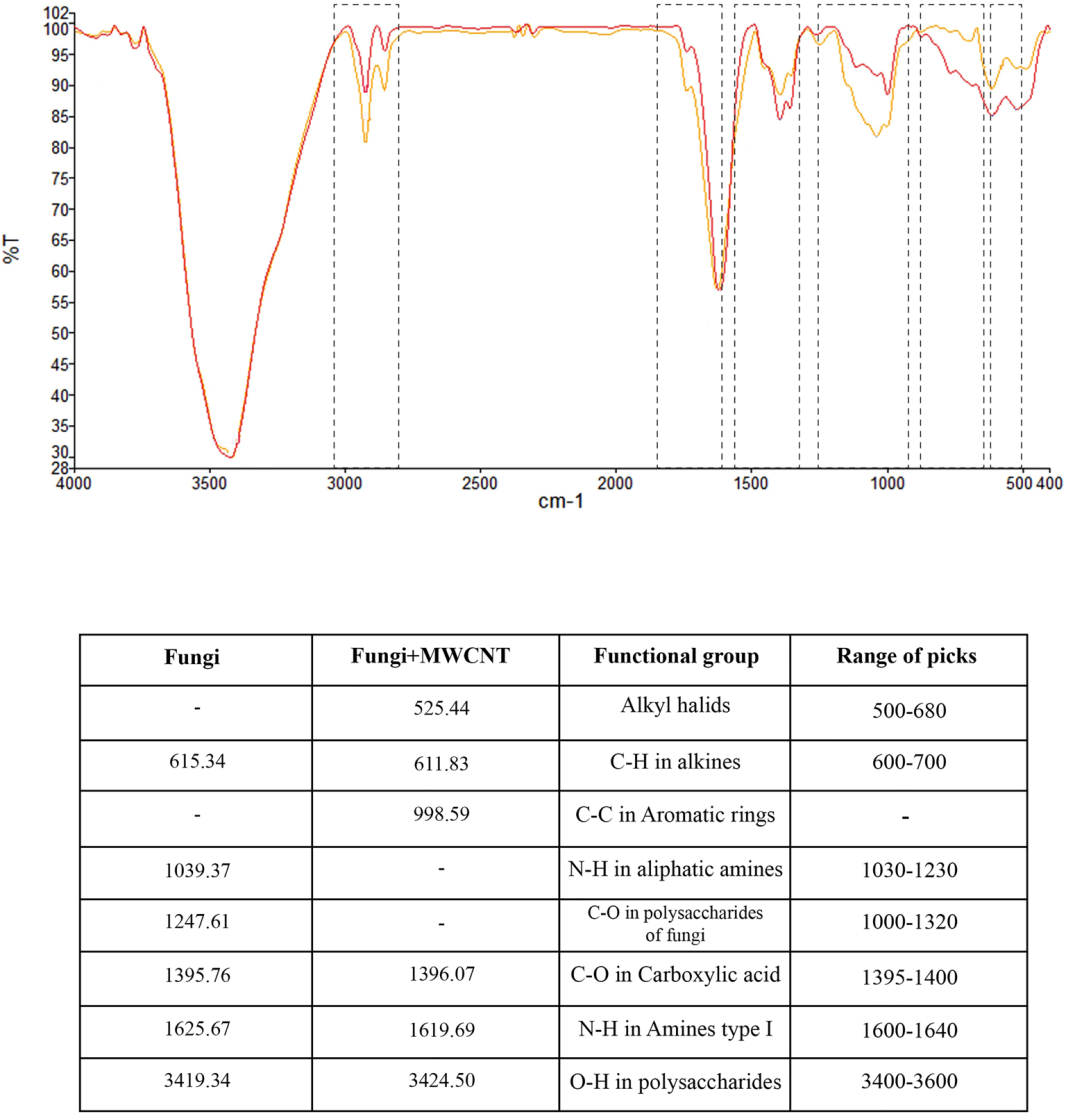
Vibrational spectra of *Trametes versicolor* fungi before and after biosorption (Yellow spectrum: Fungi, Red spectrum: Fungi + MWCNT).

Therefore, according to the main results of this analysis, it can be concluded that hydrogen interaction in amine and hydroxyl groups with an aromatic ring in carbon nanostructure (Yoshida-H binding), dipole-inter-dipole interaction between the carboxyl group in carbon nanotube and hydrogen in hydroxyl and amine functional groups, and also the n-л bond, which affects the interaction between the aromatic ring of nanocarbon and the oxygen and nitrogen groups present in the structure of the fungi are among the effective interactions in the biosorption of carbon nanotubes to the cell wall of fungi. Figure 3 shows the vibrational spectra of *T. versicolor* before and after adsorption by the CNTs and depicts the indicated shift and band positions in the wave number and variations in the FTIR functional groups.

### SEM and EDS analysis

**Figure 4:**
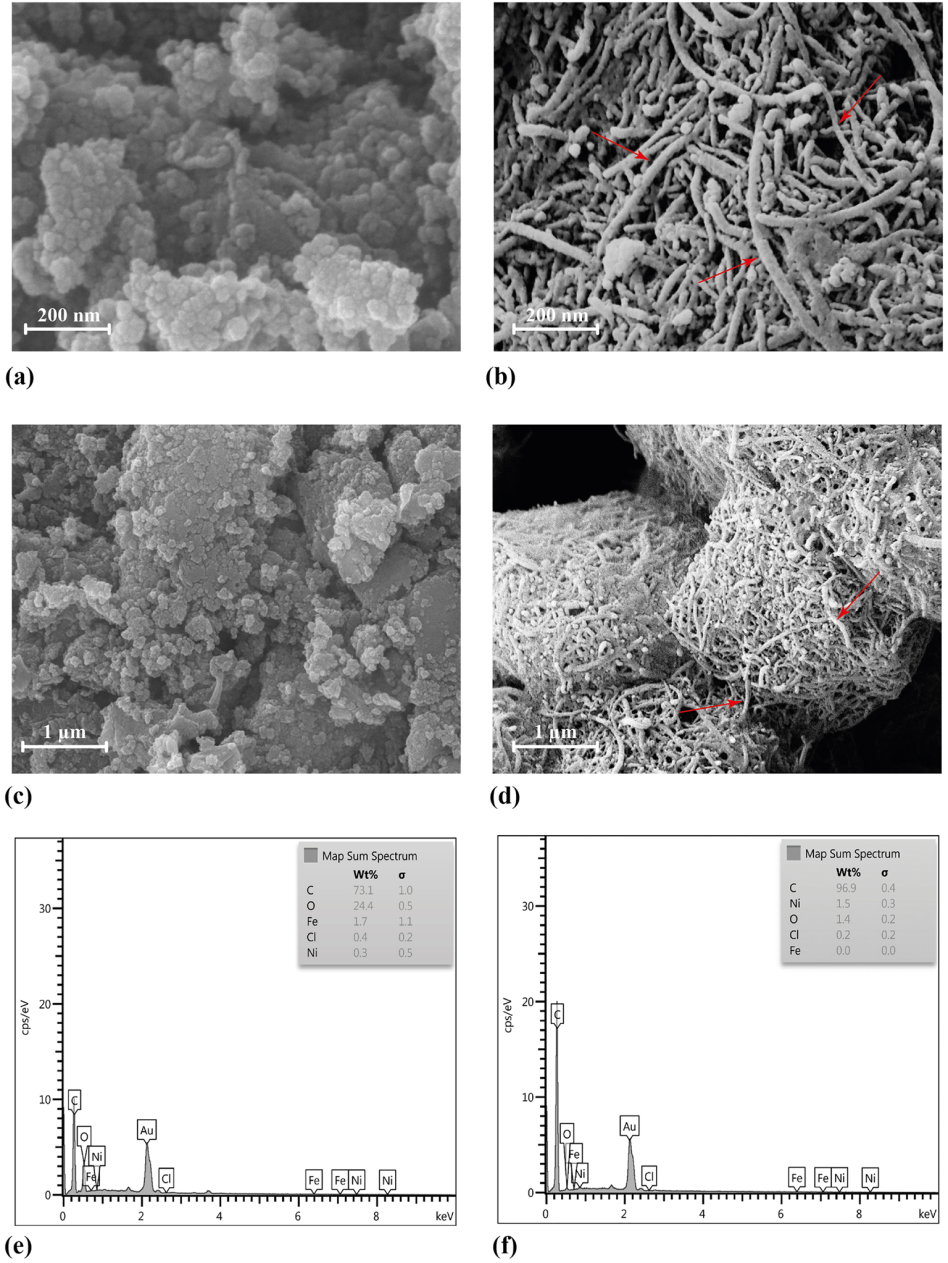
SEM photographs of fungal biosorbent before (**a**,**c**) and after (**b**,**d**) biosorption of MWCNT-COOH. EDS spectrum of fungal biosorbent before (**e**) and after (**f**) biosorption of MWCNT-COOH.

The structure and surface morphology of the MWCNT-loaded and unloaded fungal biosorbents were examined using a scanning electron microscope, as shown in Fig. 4. A comparison of two types of fungi—one containing carbon nanotubes and the other not—shows that the mycelium morphology and surface structure were conspicuously different in these two cases. In such a way that the mycelia of the fungus lack carbon nanotubes, they have a smooth surface, which becomes rough in their current state after the adsorption of carbon nanotubes. This rough and smooth state on the surface of the mycelia was visible at 1-µm magnification. In addition, at higher magnifications (200 nm), nanotube structures were observed on the surface of the mycelia.

The EDS spectra of the MWCNTs before and after biosorption onto the fungal biosorbents are shown in Fig. 4e and f. The MWCNT-COOH used in this investigation contained 98.39% C and 0.93% Ni. On the other hand, comparing the EDS spectra of the biosorbents before and after the biosorption process indicated significant changes in these elements. For example, the amount of C changed significantly from 73.1% before biosorption to 96.9% after the biosorption process. Moreover, Ni, which was the second most abundant element in the MWCNT-COOH, significantly changed from 0.3% (before biosorption) to 1.5% (after biosorption), which indicates that the increase in the amount of C and Ni is due to the addition of MWCNT-COOH on the surface of the mycelia.

### Mechanism of MWCNT-COOH removal by *T. versicolor*

The biosorption process is influenced by various chemical properties of the biosorbent, including the number of reactive binding sites, availability of binding sites, type of binding site, and structure of the biosorbate. Ion exchange, precipitation, and physical adsorption are widely recognized mechanisms of biosorption, and in some cases, the process may involve a combination of these mechanisms. The mechanisms are governed by several types of interactions, such as ionic, electrostatic, hydrogen bonding, and van der Waals forces^61^.

Ion exchange involves the exchange of ions between the adsorbate and adsorbent, where the pH of the solution plays a crucial role^61,62^. Precipitation, on the other hand, leads to the formation of insoluble particles called precipitates, which is a significant method in metabolism-dependent biosorption. In response to toxic particles in the environment, microorganisms activate a defense mechanism triggering precipitation. Interaction between functional groups on the biosorbent's cell wall and particles leads to precipitate formation^19,61^. Physical adsorption occurs on the biosorbent's surface, particularly involving cell walls in microorganisms, through interactions like van der Waals forces, with the extent of physical adsorption influenced by the biosorbent's surface area^63^.

Surface electrical charges on the biomass vary with the pH of the solution^49^. The study indicates that biosorption decreased in both acidic and alkaline pH solutions, with pH = 7 identified as the optimal pH for fungal biosorption. This suggests that the interaction between positive and negative charges, as well as ion exchange, is not the likely mechanism for nanoparticles' biosorption onto fungi cell walls. The study concludes that the mechanism for removing MWCNT-COOHs from solution using *T. versicolor* fungi is metabolism-dependent, supported by the dependency on viable cells. The process is irreversible and happens gradually over time, emphasizing its reliance on the organism's metabolic activity^26^.

Analysis of FTIR spectra before and after biosorption reveals that hydroxyl and amine groups are the primary functional groups involved in the biosorption process. These groups are found in the polysaccharides and glycoproteins of the fungal cell wall, indicating the adsorption of MWCNT-COOHs onto these structures.

**Figure 5:**
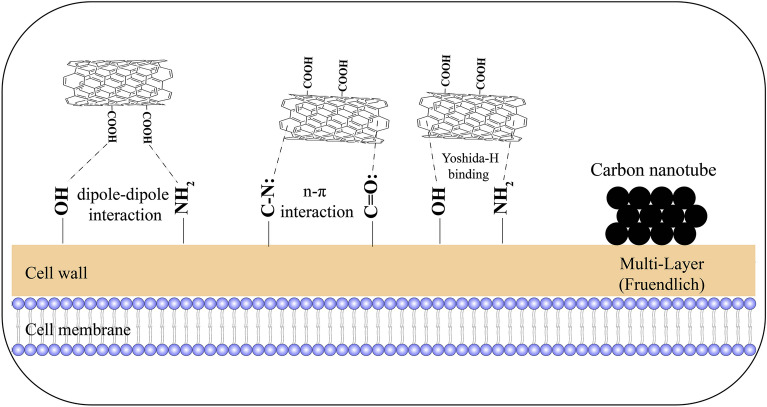
Schematic view of biosorption of MWCNTs by the cell wall of *Trametes versicolor.* (Created by Adobe Illustrator 2020, https://www.adobe.com/products/illustrator.html).

These findings suggests that the mechanism of MWCNT-COOH biosorption by *T. versicolor* fungi is a series of processes, including adsorption to the cell surface and absorption through passive and/or active transports^31^ and probably involves a combination of physical adsorption and precipitation, with potential interactions like dipole–dipole and Yoshida-H binding. Figure 5 illustrates the biosorption mechanism of MWCNT-COOH by *T. versicolor* fungi.

In general, it can be suggested that removing carbon nanotubes such as MWCNT-COOH through biosorption is an efficient and environmental-friendly method compared with other removal mechanisms. Several studies have been performed on removing carbon nanomaterials from the environment. The degradation of carbon nanomaterials, such as photodegradation in the presence of UV light, and chemical degradation in the presence of chemicals such as hydrogen peroxide, hypochlorous acid, and Fenton^21–23^. The biodegradation of carbon nanomaterials is another considerable removal mechanism. This process includes degradation by enzymes, including myeloperoxidase^64^, lignin peroxidase^65^, and lactoperoxidase^66^. Through this mechanism, carbon nanostructures are oxidized by attaching to the active site of enzymes and transferring electrons between the enzyme and carbon nanomaterials^67^.

In addition, microorganisms can influence the structure of carbon nanotubes. A study on the degradation of single-walled carbon nanotubes (SWCNT) by *T. versicolor* showed that these fungi can degrade SWCNT at a rate lower than 0.1% after 6 months of incubation^68^. In addition, a study on the potential of bacteria to degrade multi-walled carbon nanotube (MWCNT) revealed these bacteria could only degrade 0.57% of MWCNT after 3 months of incubation^69^. Investigations on the effect of microorganisms and their role in degrading carbon nanomaterials indicated that degradation by microorganisms is inefficient and, in most cases, a low degradation rate was observed. Moreover, biodegradation using enzymes is also ineffective because the process of extraction and purification is expensive. Therefore, it can be suggested that biosorption of CNTs using fungi is an efficient method that can remove considerable concentrations of CNT in a short period. Also, this method can be used in co-contaminated environments. Fungal biomass biosorbed CNTs can be used as bio-nanocomposites in removing other pollutants, such as heavy metals and dyes, due to the biosorption ability of both CNT and fungus. An example of that is a study conducted by Ding et al*.,* in which bio-nanocomposites of fungus-Fe3O4 were utilized to remove Sr(II), U(VI), and Th(IV)^70^.

## Conclusion

Carbon nanotubes are considered practical structures in various industries, including electronics and nanomedicine, owing to their high flexibility, lightweight, and electrical and heat conductivity. In this case, the concentration of these compounds is increasing. According to the reports, approximately 100 tons of products containing nanocarbon enter the environment annually, and it seems that this amount will increase in the next few years. Therefore, finding a practical solution for removing these materials from the environment is very important. Accordingly, in this experiment, the removal of MWCNT-COOHs from the environment through biosorption was introduced, and according to the results, it can be suggested as an effective way to reduce and control the amount of these recalcitrant structures in the environment and alleviate the toxicity and dangers that they may cause if they enter the environment. This study provides insights for further investigations on optimizing this method as a practical substitute for other wastewater treatment strategies for removing nanomaterials. However, factors such as culture systems, nutrient supply, and some methods for large-scale cell harvesting should be considered to use this method on a large scale.

## Data Availability

All data generated or analyzed during this study are included in this published article.
